# Capacitive field-effect biosensors loaded with intact plant virus particles: modelling and experimental data

**DOI:** 10.1007/s00216-026-06468-4

**Published:** 2026-04-01

**Authors:** Melanie Welden, Arshak Poghossian, Christina Wege, Michael J. Schöning

**Affiliations:** 1 Institute of Nano- and Biotechnologies, Aachen University of Applied Sciences, 52428 Jülich, Germany; 2 MicroNanoBio, 53125 Bonn, Germany; 3 Institute of Biomaterials and Biomolecular Systems, University of Stuttgart, 70569 Stuttgart, Germany; 4 Institute of Biological Information Processing (IBI-3), Forschungszentrum Jülich GmbH, 52425 Jülich, Germany

**Keywords:** Capacitive field-effect biosensor, Plant virus particles, *Tobacco mosaic virus*, Capacitive model, Virus coverage, Label-free detection

## Abstract

Plant viruses are considered one of the main contributors to crop losses in the agricultural industry and thus induce high economic costs worldwide. The best strategy to slow down the spread of plant virus infections and prevent crop failures is to make an early and reliable diagnosis and provide prompt treatment. Electrolyte-insulator-semiconductor capacitors (EISCAP) represent an attractive transducer architecture for label-free electrostatic detection of virus particles by their intrinsic charge. In this work, a capacitive model of an EISCAP sensor loaded with negatively charged *tobacco mosaic virus* (TMV) particles is presented. In the developed model, the adsorbed TMV particles are assumed as local gates with dimensions in the nano- to sub-micrometer range. The impact of TMV surface coverage on the sensor characteristics of SiO_2_-gate EISCAPs was studied theoretically and experimentally. The observed EISCAP signal correlates well with the coverage of loaded TMV particles evaluated from scanning electron microscopy images of the SiO_2_-gate surface.

**Graphical abstract:**

**Figure:**
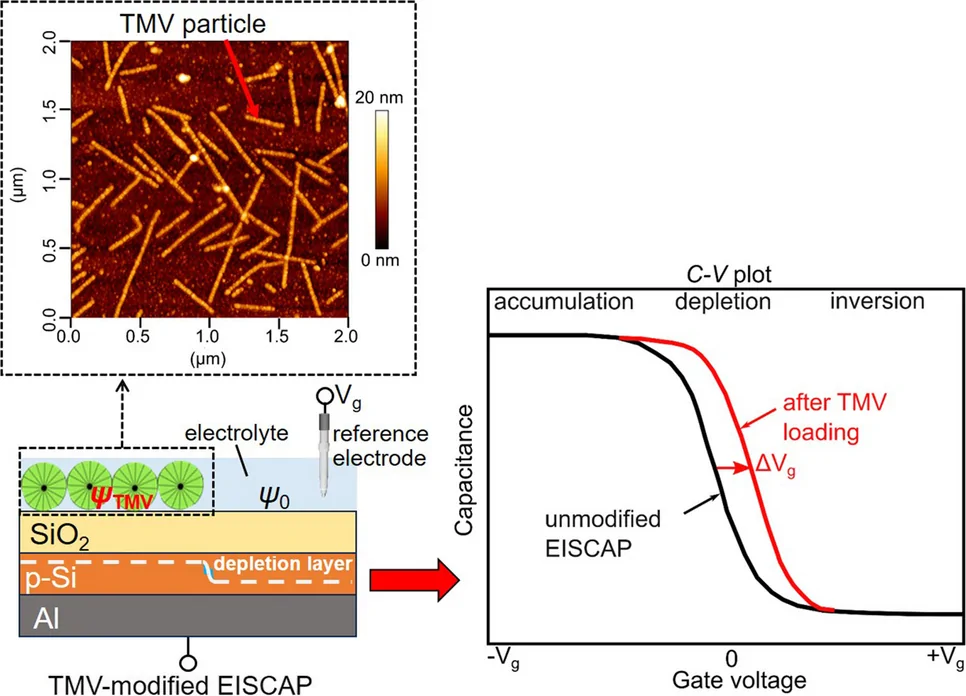

## Introduction

Plant viruses are recognized as one of the main contributors to crop losses in the agricultural industry and thus represent a serious threat to global food security. The economic losses caused by plant viruses are substantial and estimated to be between $30 and $60 billion worldwide annually, with increasing impact due to climate change and international transport of plant materials [1, 2]. Early and reliable diagnosis of plant virus infections as well as prompt treatment before disease symptoms appear is the best strategy to slow down the virus spread and prevent crop failures. Therefore, different methods and specialized tools based on the immunological, nucleic acid, and physicochemical features of viruses have been utilized for viral pathogen identification and diagnosis. Although molecular- and immunoassay-based detection technologies possess high sensitivity/specificity and represent the gold standard for providing reliable and accurate virus diagnostics, these tests are expensive, time-consuming, labor-intensive, and require skilled personnel. Moreover, they can be hardly adapted as first-line virus screening tools for on-site (i.e., in-field) applications [3–5]. Lateral-flow immunological assay (LFA) formats have recently received significant attention for point-of-care rapid virus detection [6]. LFA test strips were reported for several plant viruses, including potato virus x [7], banana bract mosaic virus [8], grapevine leafroll-associated virus [9], tobacco mosaic virus (TMV), tobacco vein banding mosaic virus, and potato virus [10]. Although LFA tests are fast, simple-to-use, and disposable, and represent relatively inexpensive diagnostic tools suitable for on-site or in-field plant virus detection, usually they have lower sensitivity and do not offer quantitative results [11].

To our knowledge, on-site-based quantitative plant virus diagnostic tests with sensitive characteristics comparable to those of laboratory diagnostic tools are currently not available. As a consequence, samples have to be collected in the field and are typically sent to qualified laboratories for analysis, which is time-consuming and cost-intensive for farmers, since many crops must be monitored repeatedly throughout the cultivation period [11]. Therefore, there is a strong interest to develop highly sensitive, accurate, rapid, cost-efficient, and portable sensor devices for in-field virus detection/identification in randomly selected plants. This should include minimal sample preparation steps and be capable of producing real-time data about viral infections in order to reduce losses in yield as well as the use of expensive, environmentally damaging chemicals. Pesticides inhibiting the transmission of viruses include insecticides, fungicides, and nematicides, all of which may have adverse effects on the host plant, humans, and the environment [12].

In contrast to highly sophisticated analytical lab tools, easily manageable biosensors could simplify the on-site plant virus diagnostics and assist virus disease management, given that they provide sufficient sensitivity and selectivity. During the last years, various biosensor-based approaches (e.g., quartz crystal microbalance [13], surface plasmon resonance [14], electrochemical impedance spectroscopy [15], capacitance measurements [16], and magnetic frequency mixing technique [11]) were proposed for detecting plant virus particles (see also recent reviews [3–5, 17]). Moreover, it has been reported on modern plant-health monitoring systems, which employ an array of various “wearable”, wireless sensors installed on the leaves of live plants and Internet of Things technologies for the transmission of measurement data indicating plant tissue invasion by viruses [18, 19].

Among different label-free sensing technologies proposed for virus detection, biosensors with semiconductor-based field-effect devices (BioFEDs) open up new possibilities: BioFEDs are not only able to identify viral antigens or nucleic acids by their intrinsic molecular charge, but also to directly electrostatically detect the adsorption/binding of charged viruses on the gate surface without the use of any label molecules [20–25]. Furthermore, BioFEDs are small in size, have a fast response time, enable real-time and multiplexed measurements, possess robustness, and are compatible with chip fabrication technologies (see, e.g., recent reviews [22, 26–28]). In the past, various types of BioFEDs were successfully implemented for the detection of diverse dangerous human and animal virus particles, such as subtypes of influenza A (H1N1 [29–31], H3N2 [30], H5N1 [31], H5N2 [32]), adenovirus [29], rotavirus [33], human immunodeficiency virus [34], Ebola [35], SARS-CoV-2 [36], etc. As indicated in [21], BioFEDs are able for ultrasensitive, label-free detection of animal and human virus particles.

On the other hand, there are only a few publications related to the detection of plant virus particles with BioFEDs. For example, plum pox virus particles were detected with extended-gate organic field-effect transistors with a lower limit of detection of 180 pg/mL [37]. Our group has, for the first time, studied the possibility of label-free electrical detection of TMV (*tobacco mosaic virus*) particles using electrolyte-insulator-semiconductor capacitors (EISCAP)—the simplest field-effect device—which is a biochemically sensitive capacitor [38, 39]. In contrast to conventional transistor-type devices, EISCAP sensors have a simple layout, which makes them easy and cost-effective in fabrication. It has been observed that the EISCAP signal is influenced by the amount of adsorbed TMV particles on the EISCAP surface [38, 39]. However, this effect has not yet been modeled, evaluated, or discussed in detail.

In this work, we present the capacitive model of an EISCAP biosensor loaded with the charged, nanorod-shaped TMV particles. The influence of surface coverage of TMV on the EISCAP response (i.e., capacitance–voltage (*C*–*V*) characteristics) has been studied. TMV was chosen as a model plant virus, because:

- it is one of the most infectious plant pathogens. It has been reported that TMVs infect more than 400 species of 36 botanical families, inducing mosaic-like patterns on the leaves and reduced growth of many hosts (e.g., pepper, tomato, aubergine, tobacco, etc.) [12], while they are non-pathogenic and non-hazardous for animals or humans [40]. In spite of a deep understanding of the TMV structure and its transmission routes, TMV causes considerable financial losses of more than 100 million dollars worldwide each year [2];
- our group has many years of expertise with enzyme-based biosensors (EISCAPs) decorated with TMV particles utilized as enzyme nanocarriers [41–45].

## Capacitive model of EISCAP covered with TMV particles

**Fig. 1:**
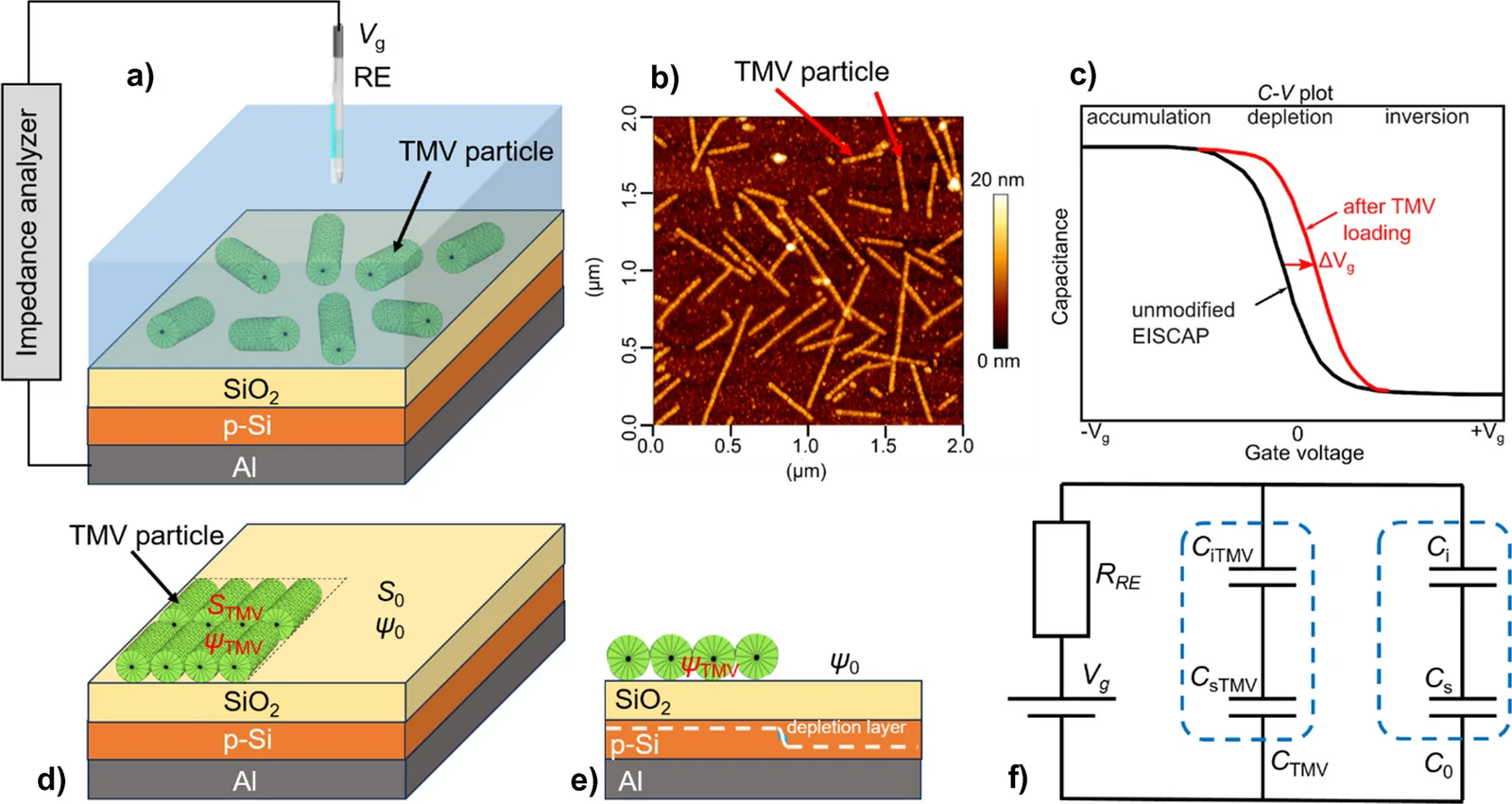
**a** EISCAP structure loaded with TMV particles (schematically); **b** AFM image of the SiO_2_-gate surface loaded with TMV particles collected in tapping mode (BioMAT Workstation, JPK Instruments, Berlin, Germany); **c** characteristic high-frequency *C–V* plot for a p-type EISCAP biosensor before and after TMV adsorption with accumulation, depletion and inversion region, respectively; **d** EISCAP surface with TMV-covered and TMV-free regions (schematically); **e** depletion layer profile in p-Si underneath regions covered with TMV and TMV-free regions (schematically); and **f** simplified electrical equivalent circuit of the TMV-covered EISCAP. RE, reference electrode; *V*_g_, gate voltage; Δ*V*_g_, shift of the *C* − *V* plot along the voltage axis;* S*_TMV_ and *S*_0_, surface areas of TMV-covered and TMV-free regions, respectively; *ψ*_TMV_ and *ψ*_0_, gate-surface potentials in TMV-covered and TMV-free regions, respectively; *R*_RE_, resistance of the RE; *C*_iTMV_, *C*_sTMV_, and *C*_TMV_, gate-insulator, depletion-layer, and overall capacitances in the TMV-covered region, respectively;* C*_i_, *C*_s_, and *C*_0_, gate-insulator, depletion-layer, and overall capacitances in the TMV-free region, respectively

The schematic structure of an EISCAP sensor chip loaded with TMV particles is illustrated in Fig. 1a. It comprises a Si substrate, which is separated from the solution by a gate-insulator film (in this work, SiO_2_), and a backside metal (Al) contact layer. Native TMV is composed of about 2130 identical coat proteins helically assembled around the single-stranded viral ribonucleic acid (see, e.g., [46]). The TMV particle has a rigid nanotube-like structure of 300 nm length complete particles, with an outer diameter of 18 nm and an inner diameter of 4 nm (nucleoprotein tube: aspect ratio ≈ 17). The isoelectric point of TMV is at pH 3.5 [47]; TMV particles are negatively charged in solutions with pH > 3.5.

Our previous experiments demonstrate that because of inter-particle repulsion as well as electrostatic and other interactions between TMV particles and the gate insulator surface, negatively charged TMV particles usually do not form an ordered and densely packed layer. This behavior is evident, for example, on SiO_2_ surfaces [39], which themselves carry a negative charge over a broad pH range (reported points of zero charge for SiO_2_ are approximately pH 2.66–2.8 [48]).

The exemplary AFM image of TMVs on the SiO_2_-gate EISCAP surface (see Fig. 1b) shows randomly distributed TMV particles with a large inter-particle spacing. Similar effects were observed, for instance, for TMV adsorbed on bare [49] or oxidized [50] Si substrates as well as on Ta_2_O_5_-gate EISCAPs [41], and may be explained by the immobility of TMV particles on the substrate surface [49]: adsorbed particles become “frozen” upon deposition, resulting in predominantly random orientation.

In addition to pH- [51, 52] and ion-sensitive [53] features, EISCAPs are known as transducer structures for designing various enzyme-based logic gates [54, 55] and as charge-sensitive devices, capable of label-free, electrical detection of adsorption/binding of various charged biomolecules (see, e.g., [56–59]) and (bio)nanoparticles (including TMV particles) on their gate surface [38, 39, 48, 60, 61]. Because of the presence of a space-charge (depletion) region nearby the semiconductor/gate-insulator interface, EISCAPs possess a variable total capacitance, which depends, among others, on the impressed gate voltage and the charge of adsorbed/bound species, such as charged TMV. Our proposed approach is based on the idea that adsorbed TMV particles represent nanometer-sized local gates, which will locally affect the space-charge distribution in the Si; in a first approximation, we neglect a possible overlapping of the local space-charge regions in the semiconductor (by, e.g., the fringing effect [62]).

For instance, in case of p-type Si, negatively charged TMV particles onto the gate-insulator surface will decrease the width of the depletion layer and increase the space-charge capacitance in the Si within regions underneath surface areas covered with TMV. As a consequence, the total capacitance of the EISCAP will increase as well, resulting in a shift of the capacitance–voltage (*C*–*V*) characteristics (one of the most convenient modes of electrochemical characterization of EISCAPs [26, 63]) in the direction of more positive (or less negative) gate voltages (Fig. 1c, red curve). The amplitude of this potential shift (Δ*V*_g_) reflects potential changes at the gate surface induced by the adsorption/binding of charged TMV particles.

Thus, owing to the operating principle of an EISCAP sensor, its response will depend on the fraction of the surface area covered with TMV particles (i.e., on the TMV surface coverage). Hence, the EISCAP loaded with charged TMV can be divided into two regions (Fig. 1d):

- a TMV-covered region with a surface area *S*_TMV_, gate-surface potential *ψ*_TMV_ and TMV coverage *θ* = *S*_TMV_/*S*, where *S* is the complete EISCAP surface area in contact with the electrolyte;
- a TMV-free region with a surface area *S*_0_ = (1 − *θ*)*S* and a gate-surface potential *ψ*_0_.

For the EISCAP application as a biosensor to detect diverse biomolecules and (bio-)nanoparticles by their intrinsic charge, the most useful range of the *C–V* plot is the depletion region (see Fig. 1c). Here, the total EISCAP capacitance is more sensitive to charge or potential changes at/nearby the gate surface. The expected change in profile of the depletion layer in the p-Si underneath the TMV-covered region is schematically depicted in Fig. 1e. To evaluate the impact of TMV coverage on the *C*–*V* curve, we derived the expression for the equivalent capacitance of the TMV-covered EISCAP in the depletion state.

The simplified electrical equivalent circuit of the TMV-covered EISCAP is illustrated in Fig. 1f. As discussed in [26, 64, 65], for a typical layer structure of the EISCAP and measuring conditions used, the resistances, such as the Al/Si backside contact, the bulk Si, the electrolyte solution and the double-layer capacitance (electrolyte/gate-insulator interface), can be neglected. Hence, the electrical equivalent circuit of a TMV-loaded EISCAP biosensor can be simplified as a parallel connection of two capacitances, *C*_0_ and *C*_TMV_ (see Fig. 1f).

The term *C*_0_ is the total capacitance associated with the TMV-free region and composed of the gate-insulator capacitance (*C*_i_ = *C*_i0_*S*(1 − *θ*)) and the depletion-layer capacitance in the Si underneath the TMV-free region (*C*_s_ = *C*_s0_*S*(1 − *θ*)) in series:

1 $${C}_{0}=\frac{{C}_{i}{C}_{s}}{{C}_{i}+{C}_{s}}=\frac{{C}_{i0}{C}_{s0}S(1-\theta )}{{C}_{i0}+{C}_{s0}}$$

where *C*_i0_ = *ε*_i_*ε*_0_/*d*_i_ is the capacitance of the gate insulator per unit surface area, *ε*_i_ and *d*_i_ are the dielectric constant and thickness of the gate insulator, respectively, *ε*_0_ is the vacuum permittivity, *C*_s0_ = *ε*_s_*ε*_0_/*w*_s_ represents the capacitance of the depletion layer in the Si per unit surface area, and *ε*_s_ and *w*_s_ are the dielectric constant of Si and width of the depletion layer, respectively. Analogously, the total capacitance *C*_TMV_ associated with the TMV-covered region is a series connection of the gate-insulator (*C*_iTMV_ = *C*_i0_*Sθ*) and the semiconductor depletion-layer (*C*_sTMV_ = *C*_s0TMV_*Sθ*) capacitance (Fig. 1f):

2 $${C}_{TMV}=\frac{{C}_{iTMV}{C}_{sTMV}}{{C}_{iTMV}+{C}_{sTMV}}=\frac{{C}_{i0}{C}_{s0TMV}S\theta }{{C}_{i0}+{C}_{s0TMV}}$$

where *C*_s0TMV_ = *ε*_s_*ε*_0_/*w*_sTMV_ and *w*_sTMV_ represent the capacitance per unit surface area and the width of the depletion layer in the Si (TMV-covered region), respectively.

The equivalent capacitance (*C*_eq_) of the whole EISCAP chip can be calculated by combining the total capacitances of the TMV-covered and TMV-free regions in parallel:

3 $${C}_{eq}{=C}_{TMV}+{C}_{0}=\frac{{C}_{i0}{C}_{s0TMV}S\theta }{{C}_{i0}+{C}_{s0TMV}}+\frac{{C}_{i0}{C}_{s0}S(1-\theta )}{{C}_{i0}+{C}_{s0}}$$

To examine the impact of the TMV coverage on the *C–V* plot of the EISCAP, we have to derive the expressions for the depletion-layer capacitances per active surface area in the Si underneath the TMV-covered and TMV-free regions (i.e., *C*_s0TMV_ and *C*_s0_). These capacitances can be deduced from the equation for the depletion capacitance of a metal–oxide–semiconductor (MOS) structure (C_s0MOS_) per unit surface area [65, 66]

4 $${C}_{s0MOS}=\frac{1}{\sqrt{\frac{1}{{C}_{i0}^{2}}+\frac{2\left({V}_{g}-{V}_{fbMOS}\right)}{q{N}_{A}{\varepsilon }_{s}{\varepsilon }_{0}}}-\frac{1}{{C}_{i0}}}$$

by replacing the flatband voltage *V*_fbMOS_ of the MOS capacitor

5 $${V}_{fbMOS}=\frac{{W}_{M}-{W}_{S}}{q}-\frac{{Q}_{i}+{Q}_{ss}}{{C}_{i0}}$$

by the flatband voltage (*V*_fb_) of the EISCAP [65, 67]:

6 $${V}_{fb}={E}_{ref}-\psi +{\upchi }_{sol}-\frac{{W}_{S}}{q}-\frac{{Q}_{i}+{Q}_{ss}}{{C}_{i0}}$$

In Eqs. (4)–(6), *V*_g_ is the gate voltage, *N*_A_ describes the density of ionized acceptors, *q* is the elementary charge,* W*_M_ and *W*_S_ are the metal and the silicon electron work functions, respectively, *Q*_i_ and *Q*_ss_ are the charges located in the oxide and the surface and interface states (for simplicity, in further description it is assumed that *Q*_i_ and *Q*_ss_ are zero), *E*_ref_ is the potential of the reference electrode, and *χ*_sol_ is the surface-dipole potential of the solution. The term *ψ* denotes the gate-surface potential: In this work, we hypothesize that *ψ* = *ψ*_0_ for the TMV-free region, *ψ* = *ψ*_TMV_ = *ψ*_0_ + Δ*ψ*_TMV_ for the TMV-covered region, and Δ*ψ*_TMV_ is the gate-surface potential change induced due to adsorbed charged TMV particles. The potential (*ψ*_0_) at the interface electrolyte/pH-sensitive gate-insulator (such as SiO_2_ used in this study) is given by [68, 69]:

7 $${\psi }_{0}=2.3\frac{kT}{q}\alpha \left({pH}_{PZC}-pH\right)$$

where *k* is the Boltzmann constant, *T* is the temperature of the solution (in K), *pH*_pzc_ represents the pH value at the point of zero charge, and *α* is the ratio of the real pH sensitivity of the gate insulator to the ideal Nernstian sensitivity (59.16 mV/pH at room temperature).

Alternatively, the capacitances *C*_s0TMV_ and *C*_s0_ can be derived from Eq. (4) by replacing *V*_g_ with the effective gate voltage (*V*_g−eff_), which is given by [68, 70]:

8 $${V}_{g-eff}={V}_{G}-{E}_{ref}+\psi -{\chi }_{sol}+{W}_{m}/q$$

resulting in the following equations for *C*_s0TMV_ and *C*_s0_:

9 $$C_{s0TMV}=\frac1{\left[1/C_{i0}^2+2\left(V_g-E_{ref}-\chi_{sol}+\psi_0{+{\triangle\psi}_{TMV}+W}_s/q\right)/q\varepsilon_s\varepsilon_0N_a\right]^{{}^{1/2}}-1/C_{i0}},$$

10 $$C_{s0}=\frac1{\lbrack{1/C_{i0}^2+2\left(V_g-E_{ref}-\chi_{sol}+{\psi_0+W}_s/q\right)/q\varepsilon_s{\varepsilon_0N}_a\rbrack}^{1/2}-1/C_{i0}}.$$

By substituting Eqs. (9) and (10) into expression (3), we obtain the following equation for the equivalent capacitance (*C*_eq_) of the TMV-loaded EISCAP in the depletion region:

11 $${C}_{eq} = S\theta \left\{\frac{1}{[{1/{C}_{i0}^{2} + 2\left({V}_{g}-{ E}_{ref}-{\chi }_{sol}+{{\psi }_{0}+{\Delta \psi }_{TMV}+W}_{s}/q\right)/q{\varepsilon }_{s}{\varepsilon }_{0}{N}_{a}]}^{1/2}}-\frac{1}{[{1/{C}_{i0}^{2} + 2\left({V}_{g }-{ E}_{ref}-{\chi }_{sol}+{{\psi }_{0}+W}_{s}/q\right)/q{\varepsilon }_{s}{{\varepsilon }_{0}N}_{a}]}^{1/2}}\right\}+\frac{S}{[{1/{C}_{i0}^{2} + 2\left({V}_{g }-{ E}_{ref}-{\chi }_{sol}+{{\psi }_{0}+W}_{s}/q\right)/q{\varepsilon }_{s}{{\varepsilon }_{0}N}_{a}]}^{1/2}}$$

Equation (11) includes two TMV-related terms, namely, the TMV coverage (*θ*) and the gate-surface potential change caused via the adsorbed charged TMV particles (Δ*ψ*_TMV_). In the depletion region, the equivalent capacitance of the TMV-loaded EISCAP linearly depends on the TMV surface coverage. A simplified relationship between Δ*ψ*_TMV_ and the effective TMV charge (*Q*_TMV_) is given by:

12 $${\Delta \psi }_{TMV}={N}_{TMV}{Q}_{TMV}/{C}_{dl}$$

where *N*_TMV_ is the theoretical maximum surface density of densely packed TMV nanotubes with an outer diameter of 18 nm and a length of 300 nm (*N*_TMV_ ~ 2 × 10^12^ TMV/cm^2^) and *C*_dl_ is the electrical double-layer capacitance per unit surface area in the TMV-covered region.

There are large discrepancies about the net surface charge of TMV particles reported in the literature [47, 71, 72], which ranges from 90 to 1800 elementary charges per virus particle dependent on the solution’s pH and ionic strength. The partial screening of the TMV charge by mobile counter ions in the analyte affects the EISCAP response, which is mainly defined by the Debye length (inversely proportional to ionic strength) and by the distance between the EISCAP surface and the effective TMV charge [73, 74]. Since TMV particles exhibit nanotubular structures, their charge is not confined directly to the EISCAP surface, but distributed over a certain distance away from the EISCAP surface. With increasing electrolyte concentration, the effectivity of electrostatic coupling between the charged TMV particles and the EISCAP surface as well as the fraction of the effective TMV charge, which will still remain inside the Debye length (i.e., detectable by the EISCAP biosensor) will decrease. Simultaneously, the double-layer capacitance (*C*_dl_) will increase. As a result, the gate-surface potential changes (Δ*ψ*_TMV_, see Eq. (12)) caused via adsorbed charged TMV particles will decrease. Hence, generally, to achieve a large biosensor signal while detecting charged TMV particles with EISCAPs, low ionic-strength electrolyte solutions are preferred that have been experimentally evidenced in [39]. If the *C–V* characterization of TMV-loaded EISCAPs is carried out in solutions with a constant pH and ionic strength (as in this study), Δ*ψ*_TMV_ can be considered approximately constant.

We have simulated the equivalent capacitance of the TMV-loaded EISCAP and *C–V* plots in the depletion region as a function of TMV coverage utilizing Eq. (11). In the model simulations, all terms in Eq. (11), except *V*_g_ and *θ*, were considered constant. The following parameters were used for the simulations: *ε*_0_ = 8.854 × 10^−12^ F/m, ε_i_ = 3.9 (SiO_2_), *d*_i_ = 30 nm, *ε*_s_ = 11.7 (Si), *S* = 50.24 mm^2^, *N*_A_ = 6.2 × 10^15^ cm^−3^ (corresponding to resistivity of a Si wafer of 2.54 Ω∙cm), *q* = 1.6 × 10^−19^ C, a group of TMV-independent potentials *V*_i-p_ = *E*_ref_ + *χ*_sol_ − *W*_s_/*q* = 0, *ψ*_0_ =  − 40 mV, *θ* = 0, 0.2, 0.4, 0.6, 0.8, and 1. For the gate-surface potential change in the region covered with adsorbed charged TMV particles, we selected a value of Δ*ψ*_TMV_ =  − 30 mV. This value is related to the results of zeta-potential (− 30.7 mV in pH 7 solution) measurements of the biotinylated TMV particles presented in [39] and was used as a working assumption for the simulations.

**Fig. 2:**
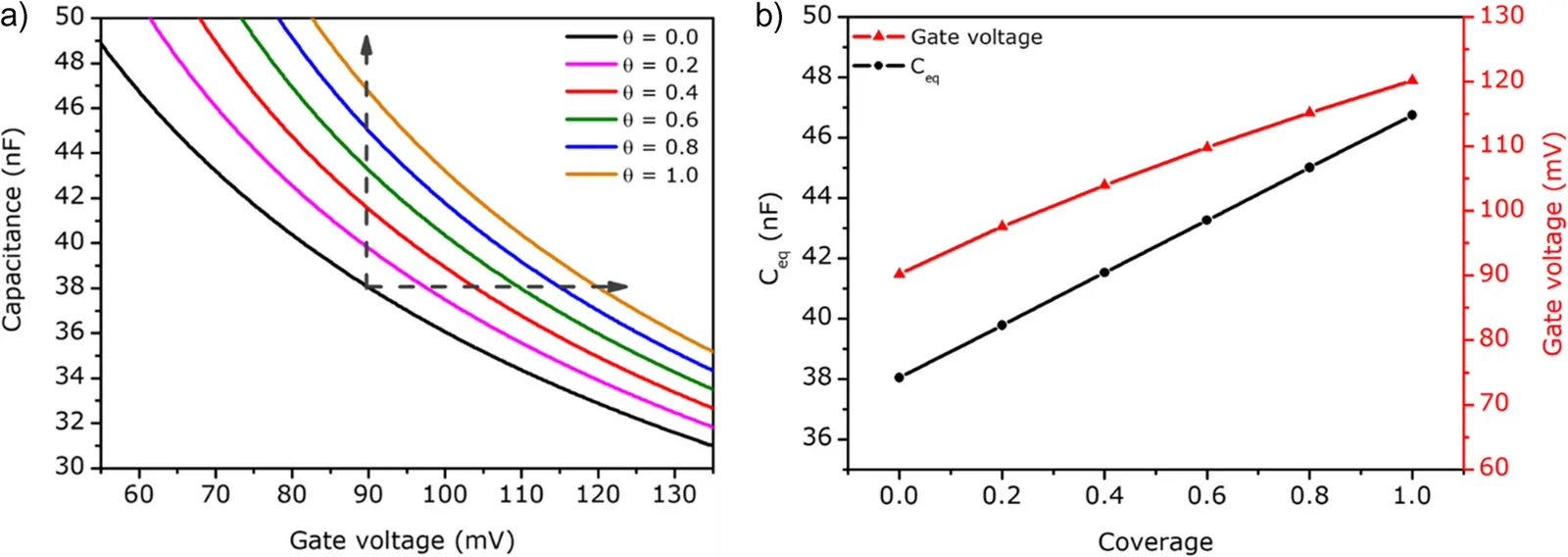
**a** Simulated *C*–*V* curves of an unmodified p-type EISCAP (black line) and an EISCAP loaded with negatively charged TMV particles with different coverages *θ*; **b** equivalent-capacitance changes (at a constant *V*_g_ = 90 mV) and voltage shifts (at a constant *C*_eq_ = 38 nF) as a function of TMV coverage, calculated from the *C–V* curves

Simulated *C*–*V* plots of a p-type EISCAP (in depletion region) without and with negatively charged TMV particles of various surface coverages are depicted in Fig. 2a. As expected, at a constant *V*_g_, loading with negatively charged TMV particles on the EISCAP surface results in an increase in the equivalent capacitance and will shift the *C–V* curve of the bare EISCAP (without TMV particles) towards more positive gate voltages. With increasing TMV coverage, the amount of the voltage shifting is increased. The equivalent-capacitance changes (at *V*_g_ = 90 mV) and voltage shifts (at *C*_eq_ = 38 nF) as a function of TMV coverage, calculated from the *C–V* curves in Fig. 2a, are illustrated in Fig. 2b.

**Fig. 3:**
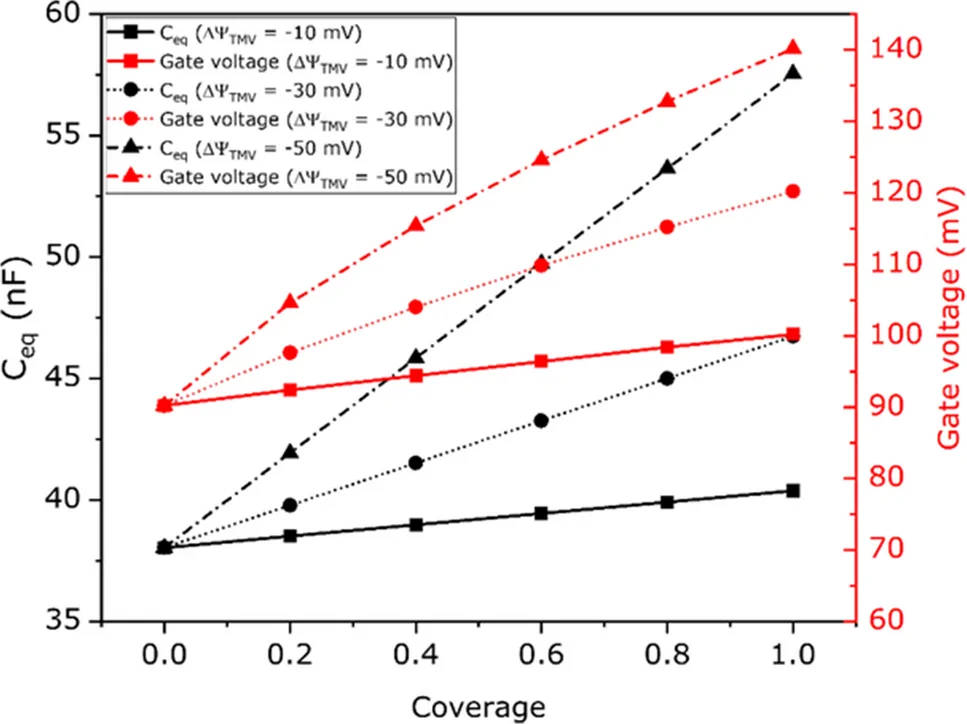
Equivalent-capacitance changes and voltage shifts as a function of TMV coverage, evaluated from the *C–V* curves simulated at various Δ*ψ*_TMV_ values of −10 mV, −30 mV, and −50 mV

It should be noted that in our simplified model, the TMV charge (*Q*_TMV_) and therefore the gate-surface potential change (Δ*ψ*_TMV_) caused via the adsorbed charged TMV particles are considered constant (at a fixed ionic strength). However, the nanotubular structure of TMV particles causes that the TMV charge is not confined directly to the sensor surface, instead being distributed through some distance (depending on Debye length) away from the EISCAP surface. On the other hand, at higher surface coverages, the TMV–TMV interactions and proximity effects may become non-negligible. All these factors may result in a more complicated distribution of both the surface potential and the profile of the depletion layer in the p-Si underneath the TMV-covered region. Therefore, to find out the impact of the amplitude of surface potential changes (or TMV charge, see Eq. (12)) on both the equivalent-capacitance changes and gate-voltage shifts, we additionally simulated the *C–V* curves at different Δ*ψ*_TMV_ values. As an example, Fig. 3 depicts the equivalent-capacitance changes and voltage shifts as a function of TMV coverage, evaluated from the *C–V* curves simulated at various Δ*ψ*_TMV_ values of −10 mV, −30 mV, and −50 mV. The analysis of Eq. (11) as well as the evaluation results in Fig. 3 reveal that at a constant TMV coverage, the raise of the Δ*ψ*_TMV_ (or *Q*_TMV_) increases the slope of both the equivalent-capacitance changes and gate-voltage shifts, resulting in an enhanced EISCAP sensitivity to TMV.

## Experimental

### Fabrication of EISCAP chips

EISCAP chips composed of a SiO_2_/p-Si/Al layer structure were fabricated from a ca. 380 µm thick p-Si wafer (Siegert Wafer, Aachen, Germany) wit <100> orientation and a specific resistivity of 1–5 Ω∙cm. A 30 nm SiO_2_ gate-insulator film was prepared onto the p-Si by thermal dry oxidation at 1000 °C for about 30 min. In the next step, the SiO_2_ layer on the backside of the p-Si was etched with 5% hydrofluoric acid, followed by electron-beam deposition of a ~300 nm thick Al layer to provide the rear-side electrical contact to the p-Si. Finally, the wafer was annealed in N_2_ atmosphere at 400 °C for 10 min and then diced into 10 mm × 10 mm single chips. Before application, the EISCAP chips were cleaned in an ultrasonic bath successively with acetone, isopropanol, ethanol and deionized water (each 5 min). Afterwards, the cleaned EISCAPs chips were mounted into a home-made measurement cell and sealed by an O-ring (Viton), protecting the side walls and the Al backside contact from the solution. The remaining front-side surface area of the SiO_2_ in contact with the solution, accessible for TMV loading, is determined via the O-ring’s inner diameter of (~8 mm) with about 50.24 mm^2^. Before electrochemical characterization of EISCAPs, the SiO_2_-gate surface was conditioned in Titrisol buffer solution of pH 7 (Merck, Germany) for at least 12 h. This procedure typically allows to achieve a more stable EISCAP signal.

### Loading of TMV particles

In this study, we used a genetically modified TMV variant with a cysteine residue exposed on each coat protein. The TMV particles were isolated from infected tobacco plants, purified and biotinylated (for details, see [42, 75]). The stock solution of TMV particles with a concentration of 5 µg/µL (corresponding to 125 nM TMV) in 10 mM sodium–potassium-phosphate buffer (SPP) prepared from 10 mM NaH_2_PO_4_ (Merck, Darmstadt, Germany) and 10 mM K_2_HPO_4_ (Carl Roth, Karlsruhe, Germany), pH 7, was stored at 4 °C.

To obtain different surface coverages, the TMV particles were loaded from differently concentrated TMV solutions. Therefore, TMV solutions with different concentrations of 20, 100, and 200 ng/µL (corresponding to 0.5, 2.5, and 5 nM) were diluted from the TMV stock solution with 100 mM SPP buffer (pH 7, ionic strength 150 mM). For TMV particle loading, 50 µL of the differently concentrated TMV solutions were drop-coated onto the SiO_2_ surface of separate EISCAP chips, installed in the appropriate measurement cells. Incubation took place at room temperature in a humid chamber. Different incubation times have been reported in the literature for loading TMV particles onto various substrates, that is, from 20 min up to 18 h (see, e.g., [38]). For example, the impact of incubation time on the density of TMV particles loaded from a 100 ng/µL TMV solution on a Ta_2_O_5_-gate EISCAP surface has been discussed in [38]. Increasing incubation time from 1 to 24 h had only a small effect on the surface density of TMV particles (~ 15%). Overall, TMV loading is nearly completed within ~ 1 h incubation time. Therefore, we decided to operate with an incubation time of 1 h. Then, the gate surfaces of the EISCAPs were rinsed with 0.33 mM phosphate-buffered saline (PBS, pH 7) to remove unattached TMV particles. The detection of the adsorbed intact TMV particles was validated by *C*–*V*-mode measurements. The pH value of all solutions was additionally controlled via a commercial pH-glass electrode (Mettler Toledo, Giessen, Germany). Overall, eight EISCAP sensors with TMV particles loaded from differently concentrated TMV solutions of 20 ng/µL (three EISCAPs), 100 ng/µL (three EISCAPs), and 200 ng/µL (two EISCAPs) were characterized.

## Results and discussion

### Field-effect detection of TMV particles by their intrinsic charge with EISCAPs operating in the *C* −* V* mode

The high-frequency *C–V* plots of EISCAPs before (bare sensor) and after loading with different concentrations of intact virus particles were recorded using an impedance analyzer (Zahner Zennium, Zahner Elektrik, Kronach, Germany). The custom-made measurement cell with the installed EISCAP chip together with the Ag/AgCl reference electrode (RE, filled with 3 M KCl, Metrohm, Filderstadt, Germany) was inserted in a Faraday cage to preclude possible impact of electromagnetic fields and ambient light influence on the EISCAP biosensor response. For C–V-mode operation, a sweeping direct-current gate voltage (between −2 V and 2 V in 0.1 V increments) and a small superimposed alternating-current voltage (0.02 V with a frequency of *f* = 120 Hz) were applied between the RE and the backside Al contact (see Fig. 1a). To reduce the influence of the Debye screening effect and to achieve a high EISCAP signal change induced by the attached TMV particles, the study was performed in a low ionic-strength (5 mM) buffer (0.33 mM PBS, pH 7, at room temperature).

In the presence of a series resistance (*R*_RE_) of the RE (other series resistances can be neglected as discussed in “Capacitive model of EISCAP covered with TMV particles”), the relationship between the measured capacitance (*C*_m_) and the equivalent capacitance (*C*_eq_) is defined by [76]:

13 $${C}_{m}=\frac{{C}_{eq}}{1+{(2\pi f{R}_{RE}{C}_{eq})}^{2}}$$

For our experimental conditions, the term (2π*fR*_RE_*C*_eq_)^2^ <  < 1. Hence, the measured capacitance can be considered approximately equal to the equivalent capacitance, *C*_m_ ≈ *C*_eq_.

**Fig. 4:**
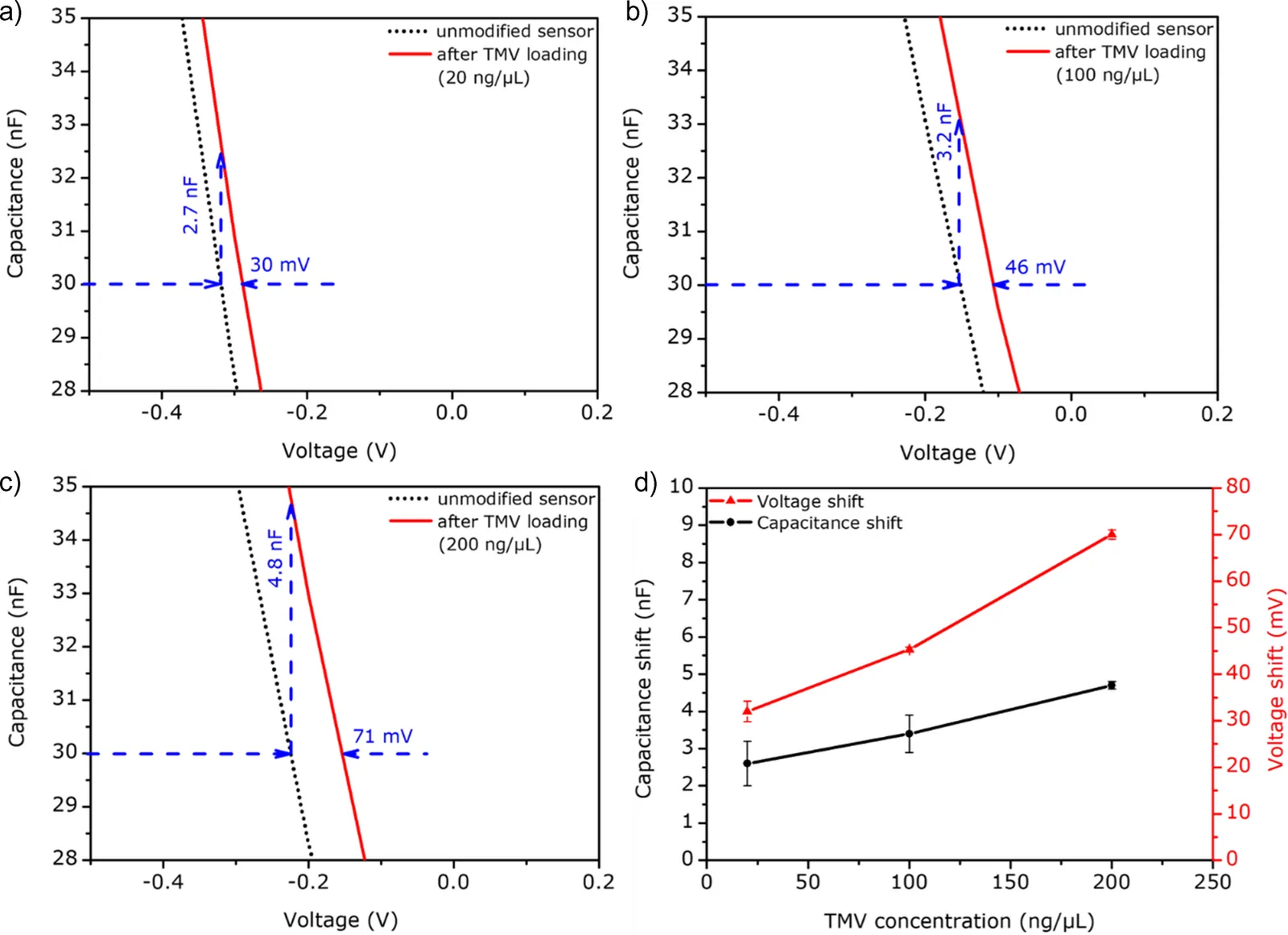
Exemplary *C–V* plots of EISCAPs in the depletion region (zoom-in for applied gate voltages between −0.5 and 0.2 V) recorded before and after loading with different concentrations of TMV particles: **a** 20 ng/µL, **b** 100 ng/µL, **c** 200 ng/µL; **d** mean changes in the measured total capacitance (black line) of the TMV-covered EISCAPs in the depletion region and the mean amplitude (red line) of the voltage shifts (at a constant capacitance of 30 nF) as a function of TMV concentration in solution evaluated from the respective *C*–*V* plots

Figure 4 depicts exemplary *C–V* plots of EISCAP sensors in the depletion region recorded before and after loading with different concentrations of TMV particles (Fig. 4a–c). As expected, the adsorption of charged virus particles on the EISCAP surface resulted in a TMV concentration-dependent shift of the original (without TMV particles) *C–V* plot along the voltage axis. The direction of these shifts corresponds to more negatively charged gate surfaces (see also discussion in “Capacitive model of EISCAP covered with TMV particles”) indicating the successful loading of negatively charged TMV particles on the sensor chip. At the same time, the amplitude of the signal shifts gives information about the surface coverage and amount of adsorbed virus particles per EISCAP chip and therefore allows for the establishment of a relationship between the TMV concentration in solution and the TMV coverage. With rising TMV concentration from 20 to 200 ng/µL, both the measured capacitance change and the shift of the *C*–*V* plot due to the adsorption of TMV particles increased from 2.7 to 4.8 nF and from 30 to 71 mV, respectively. Remarkably, even the lowest TMV concentration of 20 ng/µL was detectable with the EISCAP biosensor, having a signal of 30 mV.

The mean changes in the measured total capacitance of the TMV-covered EISCAPs in the depletion region (at a constant gate voltage corresponding to a capacitance of 30 nF for the unmodified EISCAP) and the mean amplitude of the voltage shifts (at a constant capacitance of 30 nF), calculated from the respective *C*–*V* plots, are depicted in Fig. 4d. The mean TMV sensitivity of SiO_2_-gate EISCAPs amounted to 35 ± 1.4 mV/dec (or about 2.1 nF/dec) in the TMV concentration range from 20 to 200 ng/µL (0.5–5 nM). The lower detection limit was estimated according to Ref. [77] by the intersection between the calibration curve and the mean background signal plus threefold background standard deviation. The evaluation of the detection limit for TMV particles using the experimental data presented in Fig. 4d reveals that EISCAPs are able to detect TMV particles as low as ~ 1 ng/µL.

### Impact of TMV surface coverage on the EISCAP signal

To examine the impact of TMV coverage on the EISCAP signal, SEM images were collected from the active surface area (in contact with solution) of EISCAPs loaded from differently concentrated TMV solutions. Therefore, after *C*–*V* characterization, the TMV-decorated EISCAPs were demounted from the measurement cell, washed with deionized water, and prepared for SEM characterization. SEM images were taken from six representative areas of each EISCAP chip surface using a high-resolution Jeol JSM-7800F Schottky field-emission microscope (JEOL GmbH, Freising, Germany).

**Fig. 5:**
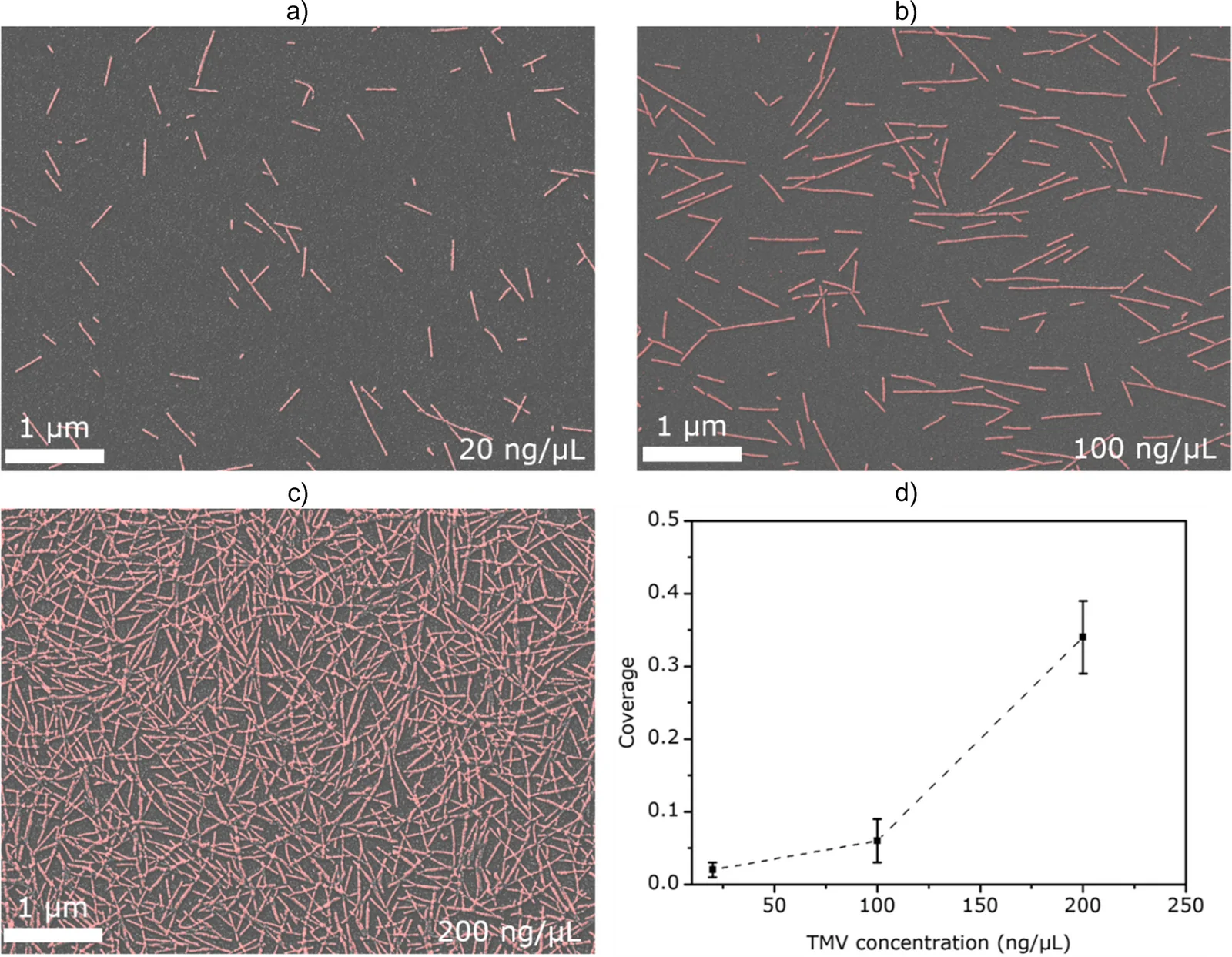
Exemplary SEM images of EISCAP surfaces loaded with TMV particles from differently concentrated TMV solutions: **a** 20 ng/µL, **b** 100 ng/µL, and **c** 200 ng/µL; **d** mean TMV coverage on the SiO_2_-gate EISCAP surfaces evaluated from SEM images (n = 6 representative areas of each EISCAP chip surface) as a function of TMV concentration in solution

Figure 5 shows SEM images of EISCAP surfaces with loaded TMV particles from differently concentrated TMV solutions (20 to 200 ng/µL). In the SEM images, randomly oriented, single complete 300 nm long TMV particles, longer particles of more than 600 nm formed by end-to-end aggregation as well as disintegrated shorter TMV fragments or particle intermediates (having lengths from 50 to 200 nm) are clearly visible. In addition, the virus network contained complete and/or incomplete TMVs, which randomly overlapped with each other, thereby forming a partial bilayer or jackstraws configuration rather than a monolayer (see, e.g., SEM image in Fig. 5c). A higher number of adsorbed TMV particles (and therefore, an increased surface coverage) was found with higher TMV concentration in solution. This corresponds to results when loading TMV particles on a gold-covered Si wafer [78] and a Ta_2_O_5_ surface [38].

To determine the density of TMV particles on the EISCAP surface from SEM images, in our previous experiments, the number of differently sized as well as overlapped virus particles was multiplied by several predetermined correction factors [38, 39, 45]. Then, the evaluated number of TMV particles per EISCAP chip was used to calculate the surface coverage of densely packed TMV nanotubes with an outer diameter of 18 nm and a length of 300 nm. However, such an approach may provoke distinct errors with regard to the real surface coverage of TMV particles and evaluation of the EISCAP signal.

In the present study, we used a proprietary Python-based program to quantify the TMV coverage on the EISCAP surface. The algorithm identifies TMV-covered regions based on image contrast and labels these areas accordingly (displayed as red in the SEM images, Fig. 5). The surface coverage is then calculated from the ratio of the labeled area to the total imaged area. This method allows direct determination of the TMV surface coverage more accurately and without the use of various correction factors for calculating differently sized TMV particles.

The mean TMV coverage on the SiO_2_-gate EISCAP surfaces evaluated from SEM images (n = 6 representative areas of each EISCAP chip) as a function of TMV concentration in solution is depicted in Fig. 5d. With increasing the TMV concentration in solution from 20 ng/µL to 200 ng/µL, the mean surface coverage increased from *θ* = 0.02 ± 0.01 to *θ* = 0.34 ± 0.05 (maximum mean TMV coverage achieved in this study).

Table 1 illustrates the correlation between the TMV coverage and the EISCAP signal change, i.e., the gate-voltage and capacitance shift, respectively. As predicted by the capacitive model described in “Capacitive model of EISCAP covered with TMV particles”, the higher the TMV coverage, the larger are the recorded *C–V* signal shifts induced by the adsorbed TMV particles. For example, with increasing the TMV coverage from 0.02 to 0.34, the amplitude of mean signal shifts raises from 32 to 70 mV (gate-voltage shift) or 2.6 nF to 4.7 nF (measured capacitance change).

**Table 1:** Correlation between the mean TMV surface coverage (*θ*) and mean EISCAP signal changes: gate-voltage (Δ*V*_g_) and measured capacitance (Δ*C*) shifts, respectively

*θ*	Δ*V*_g_ (mV)	Δ*C* (nF)
0.02 ± 0.01	32 ± 2	2.6 ± 0.6
0.06 ± 0.03	45 ± 0.5	3.4 ± 0.5
0.34 ± 0.05	70 ± 1	4.7 ± 0.1

The quantitative comparison of simulated and experimental results seems to be very difficult because Eq. (11) includes some parameters where the values are not exactly known by experimental conditions used in this work (e.g., the doping concentration in Si (*N*_A_), gate-surface potential change caused via the adsorbed charged TMV particles (Δ*ψ*_TMV_), effective TMV charge (*Q*_TMV_), electrical double-layer capacitance per unit surface area in the TMV-covered region (*C*_dl_), and surface-dipole potential of the solution (χ_sol_)).

## Conclusions

A capacitive model of an EISCAP sensor loaded with negatively charged TMV particles as a model plant pathogen is presented, in which the adsorbed TMV particles are assumed as local gates in the nanometer size range that locally affect the space-charge distribution and depletion capacitance in the Si. The impact of TMV surface coverage on the *C*–*V* characteristics of Al-Si-SiO_2_ EISCAPs was studied theoretically and experimentally. Different TMV coverages (*θ* = 0.02, 0.06, and 0.34) on the EISCAP gate surface were achieved by means of TMV loading from differently concentrated TMV solutions of 20, 100, and 200 ng/µL, respectively. The mean surface coverage of TMV particles was evaluated from SEM images for six representative areas of each EISCAP chip surface using a proprietary Python-based program.

Both the simulation and the experimental results reveal that there is a good correlation between the TMV coverage and the EISCAP signal change. While raising the coverage of negatively charged TMV particles, both the measured capacitance changes (at a constant *V*_g_) and the shifts of the original *C–V* curve (at a constant capacitance) towards more positive (or less negative) gate voltages increased. The EISCAP biosensor can detect TMV particles with concentrations as low as 20 ng/µL, having a mean signal amplitude of 30 mV.

The obtained results demonstrate significant influence of the virus surface coverage on the EISCAP biosensor response, which should be taken into account while developing EISCAP-based quantitative plant virus detectors. In future work, the developed model for the EISCAP sensor loaded with TMV particles will be extended to also consider the impact of overlapping TMV particles and depletion regions as well as the charge-screening and fringing effects on the EISCAP signal. Such a procedure will allow us to gain deeper insight into this novel sensing platform for a label-free electrostatic detection of viruses.

## Data Availability

Data are available upon request from the authors.

## Code availability

Code is available upon request from the authors.
